# Chrononutrition and Pediatric Metabolic Health: An Integrative Review of Circadian Biology and Early-Day Metabolic Regulation

**DOI:** 10.7759/cureus.113864

**Published:** 2026-08-03

**Authors:** Bruno Costa

**Affiliations:** 1 Nutrition, Private Practice, Santos, BRA

**Keywords:** child health, chronobiology, chrononutrition, circadian rhythm, early-day metabolic window, feeding behavior, metabolism, pediatric metabolism, pediatric obesity

## Abstract

Childhood obesity remains one of the most important public health challenges worldwide, resulting from the complex interaction of biological, behavioral, environmental, and socioeconomic factors. Although substantial advances have been achieved in nutritional science, increasing evidence suggests that metabolic regulation is influenced not only by dietary composition but also by biological timing. This narrative conceptual review synthesizes current knowledge from chronobiology, chrononutrition, pediatric endocrinology, nutritional physiology, and behavioral nutrition to examine how early-day eating patterns may influence metabolic regulation during childhood.

The available evidence indicates that metabolic responses emerge from the coordinated interaction among circadian organization, endocrine signaling, dietary quality, feeding behavior, and developmental physiology rather than from isolated nutritional variables alone. Based on this integrated interpretation, this review proposes the concept of the Early-Day Metabolic Window (EMW), defined as the physiological period immediately following overnight fasting during which circadian synchronization, endocrine responsiveness, metabolic flexibility, and nutrient sensing converge during the transition toward daytime metabolism.

Building upon this concept, we present the Costa Reset Conceptual Model, an integrative physiological framework designed to organize current evidence into a unified interpretation of early-day metabolic regulation. Rather than proposing a new biological mechanism or a specific dietary intervention, the model provides a conceptual structure through which biological timing and nutritional exposures may be interpreted as interacting determinants of pediatric metabolic regulation.

Although the proposed framework remains conceptual and requires prospective experimental validation, it generates biologically plausible and testable hypotheses that may guide future clinical and translational research. Integrating chrononutrition and temporal nutritional physiology into pediatric nutritional science may contribute to a more comprehensive understanding of childhood metabolic health and support future chrononutrition-based preventive and therapeutic strategies.

## Introduction and background

Childhood obesity has emerged as one of the most critical public health challenges of the 21st century, with prevalence rates increasing at an alarming pace across countries of all income levels [1-3]. Once considered a problem primarily affecting high-income nations, pediatric obesity has become a truly global epidemic, driven by profound changes in dietary habits, urbanization, sedentary lifestyles, sleep behavior, and the widespread availability of energy-dense, ultra-processed foods [1,2]. The World Health Organization estimates that hundreds of millions of children and adolescents worldwide are currently living with overweight or obesity, highlighting an unprecedented epidemiological transition with substantial implications for healthcare systems, economic sustainability, and population health over the coming decades [1].

The clinical consequences of childhood obesity extend far beyond excessive adiposity [3,4]. Excess body weight acquired during early life is strongly associated with the premature development of metabolic abnormalities, including insulin resistance, impaired glucose tolerance, dyslipidemia, elevated blood pressure, chronic low-grade inflammation, non-alcoholic fatty liver disease, and endothelial dysfunction [3,4]. Importantly, these alterations frequently emerge before adulthood and tend to persist throughout the life course, significantly increasing the long-term risk of type 2 diabetes mellitus, cardiovascular disease, and premature mortality [5,6]. Increasing evidence also indicates that obesity during childhood may induce metabolic adaptations that become progressively more difficult to reverse as physiological systems mature, reinforcing the importance of preventive interventions during the earliest stages of life [3,5,6].

Although excessive caloric intake and reduced energy expenditure remain fundamental determinants of positive energy balance, contemporary evidence indicates that pediatric obesity cannot be adequately explained through the traditional paradigm of "calories consumed versus calories expended" [3,7]. Instead, body weight regulation is increasingly recognized as the result of a complex interaction among genetic susceptibility, endocrine signaling, behavioral patterns, environmental influences, developmental programming, and metabolic adaptation [3,7,8]. This multidimensional perspective has encouraged researchers to investigate biological mechanisms capable of influencing metabolic efficiency independently of total caloric intake, thereby expanding the understanding of obesity beyond quantitative aspects of nutrition alone [7,8].

Among the emerging concepts that have transformed nutritional science over the past decade, chrononutrition has attracted considerable attention for proposing that the physiological effects of food consumption depend not only on nutrient composition and energy content but also on the timing of food intake in relation to the body's endogenous circadian organization [9-11]. This concept challenges the conventional assumption that identical meals exert equivalent metabolic effects regardless of when they are consumed [7,9]. Instead, accumulating evidence suggests that nutrient utilization, hormonal responses, substrate oxidation, appetite regulation, and postprandial glucose metabolism exhibit substantial circadian variation throughout the 24-hour day, indicating that meal timing constitutes an independent determinant of metabolic health [7,9-11].

Circadian rhythms are generated by an evolutionarily conserved biological timing system that coordinates physiological and behavioral functions according to the environmental light-dark cycle [7,12]. In mammals, this temporal organization is orchestrated by the suprachiasmatic nucleus of the anterior hypothalamus, which synchronizes peripheral molecular clocks distributed throughout virtually all metabolically active tissues, including the liver, pancreas, skeletal muscle, adipose tissue, gastrointestinal tract, and endocrine organs [12,13]. These peripheral oscillators regulate the rhythmic expression of thousands of genes involved in glucose homeostasis, lipid metabolism, mitochondrial function, inflammatory pathways, hormonal secretion, and cellular energy balance, thereby optimizing metabolic processes according to predictable daily environmental demands [13,14].

The molecular architecture of the circadian system is maintained through interconnected transcriptional-translational feedback loops involving highly conserved clock genes, including CLOCK, BMAL1, PER, and CRY, whose coordinated oscillatory activity regulates the temporal expression of numerous downstream metabolic pathways [14,15]. Environmental stimuli, known as zeitgebers, continuously synchronize these molecular clocks to maintain circadian alignment [13,15]. While the light-dark cycle remains the dominant synchronizing signal for the central clock, feeding behavior has emerged as one of the most powerful zeitgebers influencing peripheral metabolic clocks [13,16]. Consequently, the timing, frequency, and regularity of food intake have become recognized as critical determinants of circadian synchronization, capable of modulating metabolic responses independently of dietary composition [16].

Growing evidence indicates that disruption of circadian organization may have profound metabolic consequences [16-18]. Experimental and observational studies have consistently demonstrated that circadian misalignment resulting from irregular sleep schedules, prolonged eating windows, late-night food intake, shift work, or inconsistent meal timing impairs insulin sensitivity, alters glucose metabolism, disrupts hormonal secretion, promotes systemic inflammation, and increases the risk of obesity and cardiometabolic disease [17,18]. Importantly, these alterations appear to occur even in the absence of significant differences in total caloric intake, suggesting that temporal organization itself represents an essential component of metabolic regulation [16,17]. Such findings have progressively shifted the scientific discussion from the exclusive evaluation of dietary quantity toward a broader understanding of how biological timing interacts with nutritional behavior to influence human metabolism [7,16].

Over the past two decades, chrononutrition has evolved from a theoretical concept into a rapidly expanding field of nutritional science that investigates the reciprocal interactions between biological timing and dietary behavior [9-11]. Rather than considering nutrition exclusively through the quantitative dimensions of caloric intake or nutrient composition, chrononutrition recognizes food intake as a temporal signal capable of interacting with endogenous circadian rhythms and influencing metabolic homeostasis [9-11,19]. This emerging perspective has broadened the understanding of nutritional physiology by demonstrating that identical dietary patterns may produce distinct metabolic responses depending on the biological time at which food is consumed [19,20], emphasizing meal timing as an independent determinant of health outcomes [10,20].

Human metabolism exhibits pronounced circadian oscillations that influence multiple physiological processes throughout the day [7,16]. Glucose tolerance, insulin sensitivity, pancreatic β-cell responsiveness, hepatic glucose production, lipid oxidation, diet-induced thermogenesis, gastric emptying, and appetite regulation all vary according to circadian phase [19,21]. These rhythmic fluctuations reflect the coordinated activity of central and peripheral biological clocks, allowing metabolic pathways to anticipate predictable environmental demands rather than merely reacting to them [7,16,22]. Consequently, nutrient metabolism is not biologically constant over a 24-hour period, and identical meals consumed at different times may elicit substantially different endocrine and metabolic responses [19,21,22].

Evidence accumulated from controlled laboratory experiments, observational studies, and clinical interventions consistently suggests that meal timing influences metabolic efficiency independently of dietary composition [20]. Individuals consuming a greater proportion of their daily energy intake earlier in the day generally demonstrate improved glycemic control, enhanced insulin sensitivity, greater postprandial metabolic flexibility, and more favorable body weight regulation compared with those whose caloric intake is concentrated during evening or nighttime hours [21,23-25]. Conversely, delayed eating patterns have been associated with impaired glucose metabolism, increased adiposity, reduced diet-induced thermogenesis, and greater cardiometabolic risk, even when total daily energy intake remains comparable [21,24-26], reinforcing the concept that meal timing exerts independent metabolic effects beyond caloric quantity alone [20,26].

These findings have stimulated increasing scientific interest in dietary strategies designed to optimize metabolic health through temporal organization of food intake [20,26]. Time-restricted eating, early time-restricted feeding, circadian fasting protocols, and interventions based on meal timing have demonstrated promising metabolic effects in several adult populations, including improvements in insulin sensitivity, blood pressure, inflammatory biomarkers, and body composition [23-27]. Although the majority of intervention studies have been conducted in adult populations, the physiological mechanisms underlying these observations are highly relevant to pediatric metabolism, particularly because endocrine and circadian systems are established early in life [24,26,27].

Despite the considerable expansion of chrononutrition research, mechanistic and interventional evidence specifically involving pediatric populations remains substantially more limited than that available for adults [20]. Childhood represents a unique developmental period characterized by continuous physiological maturation, endocrine adaptation, rapid somatic growth, and remarkable metabolic plasticity [3,28]. During this stage, environmental influences, including dietary habits, sleep patterns, physical activity, and family routines, may exert long-lasting effects on metabolic programming that extend well into adulthood [3,28]. Consequently, understanding how circadian biology interacts with nutritional behavior during childhood has become an increasingly relevant research priority, particularly in light of the growing prevalence of pediatric obesity worldwide [20,28,29].

Among the behavioral components receiving growing scientific attention, the first nutritional exposure following the overnight fasting period has emerged as a potentially important determinant of metabolic regulation [19,21,22]. Morning physiology is characterized by complex endocrine adaptations that include activation of the hypothalamic-pituitary-adrenal axis, the cortisol awakening response, progressive changes in insulin sensitivity, modulation of incretin secretion, and coordinated activation of peripheral metabolic pathways [19,22,30]. These physiological events suggest that the metabolic consequences of the first meal of the day may extend beyond immediate postprandial responses, potentially influencing appetite regulation, substrate utilization, energy balance, and subsequent food choices throughout the remainder of the day [30,31].

However, profound transformations in modern dietary behavior have substantially modified the nutritional characteristics of breakfast in many populations [32,33]. Traditional breakfasts based on minimally processed foods have progressively been replaced by highly processed products characterized by refined carbohydrates, added sugars, industrial fats, low dietary fiber content, artificial additives, and high glycemic load [34-37]. This nutritional transition has occurred alongside increasing breakfast skipping, irregular eating schedules, prolonged daily eating windows, and greater reliance on convenience foods, particularly among children and adolescents [32,33]. Such behavioral changes have raised important questions regarding the interaction between meal timing, dietary quality, and circadian physiology during a critical period of metabolic regulation [20,34,37].

The metabolic implications of these dietary patterns extend beyond simple caloric excess [34-37]. Ultra-processed foods are typically characterized by high energy density, elevated glycemic load, reduced satiety potential, lower micronutrient density, and physicochemical properties that may facilitate excessive energy consumption [34-37]. Experimental evidence further suggests that these foods may influence hormonal regulation, inflammatory pathways, gut microbiota composition, and reward-related neural mechanisms involved in appetite control [35-37]. When consumed during periods of heightened metabolic responsiveness, such as the early hours following awakening, these dietary characteristics may interact with endogenous circadian physiology, potentially amplifying their effects on endocrine regulation and metabolic homeostasis [31,37].

Collectively, these observations indicate that metabolic health cannot be fully explained by isolated nutritional variables such as caloric intake, macronutrient distribution, or meal frequency alone [7,9]. Rather, an integrated understanding requires consideration of how biological timing, dietary quality, endocrine regulation, and behavioral patterns interact throughout the day [16,20]. Although substantial progress has been achieved in each of these individual research areas, important conceptual gaps remain regarding the integration of these mechanisms within the specific context of early-day eating behavior during childhood, thereby supporting the development of integrative conceptual frameworks capable of guiding future mechanistic and clinical investigations [9,20].

Although the scientific literature has substantially advanced the understanding of chrononutrition, several important questions remain unresolved, particularly regarding pediatric metabolic physiology [10,20,29]. Most available evidence has examined isolated components of dietary behavior, including breakfast consumption, meal frequency, eating duration, time-restricted eating, and overall dietary quality, providing valuable insights into nutritional physiology but offering relatively limited integration of these mechanisms within unified conceptual frameworks, particularly in pediatric populations [20,29,38,39].

Current evidence regarding breakfast consumption illustrates this complexity [33,40]. For decades, breakfast has traditionally been promoted as the most important meal of the day, largely based on observational studies reporting associations between regular breakfast consumption and lower body mass index, improved dietary quality, enhanced academic performance, and reduced cardiometabolic risk [33,41,42]. However, more recent investigations have challenged the universality of these conclusions by demonstrating considerable heterogeneity among study populations, methodological approaches, and definitions of breakfast itself [40,42]. These inconsistencies suggest that the metabolic effects attributed to breakfast may depend on its timing, nutritional composition, degree of food processing, and the physiological context in which the first daily nutritional exposure occurs [33,40].

Indeed, growing evidence indicates that breakfast quality, macronutrient composition, glycemic characteristics, degree of food processing, meal timing, sleep duration, circadian alignment, habitual eating behavior, and overall lifestyle may all influence metabolic outcomes [20,22,31,33,34]. Consequently, breakfast should no longer be regarded as a homogeneous nutritional intervention but rather as a complex behavioral event whose physiological consequences likely depend on multiple interacting biological and environmental factors [10,20,33]. This perspective has progressively shifted scientific attention from the simplistic question of whether breakfast should be consumed toward a broader investigation of how the characteristics of the first eating episode influence metabolic regulation [10,20,40].

This conceptual evolution is particularly relevant in pediatric populations [20,29]. Childhood represents a period of remarkable developmental plasticity during which metabolic pathways, endocrine regulation, eating behavior, and circadian organization continue to mature [3,28]. Environmental exposures during these early stages may produce adaptive responses capable of influencing long-term metabolic trajectories through mechanisms involving hormonal regulation, energy homeostasis, inflammatory signaling, and behavioral conditioning [3,28]. Consequently, nutritional interventions implemented during childhood may generate physiological effects that differ substantially from those observed in adults, reinforcing the need for pediatric-specific conceptual models [20,29].

Another important limitation within the current literature is the relative scarcity of integrative approaches capable of simultaneously considering circadian biology, dietary composition, behavioral nutrition, and metabolic physiology [20,38]. Most investigations have focused on isolated associations between individual variables, whereas the biological systems regulating energy metabolism operate through highly interconnected and dynamic networks [7,16]. Understanding pediatric metabolic health therefore requires moving beyond reductionist interpretations toward frameworks that acknowledge the continuous interaction between environmental cues, endocrine regulation, molecular circadian mechanisms, and dietary behavior throughout the daily cycle [16,20,38].

Recent advances in systems biology, nutritional endocrinology, and chronomedicine increasingly support this integrative perspective [16,20,38]. Rather than functioning as independent determinants, sleep behavior, light exposure, physical activity, meal timing, dietary quality, and feeding frequency appear to interact synergistically to influence metabolic homeostasis [7,16,38]. Within this context, the first eating episode following the overnight fasting period represents a particularly intriguing physiological event because it occurs during a phase characterized by coordinated endocrine activation, circadian synchronization, and metabolic transition from the fasting to the postprandial state [20,22,43]. Nevertheless, the mechanisms through which these processes collectively influence pediatric metabolic regulation remain incompletely characterized [20,38,43].

The growing burden of childhood obesity further reinforces the clinical relevance of addressing these knowledge gaps [3]. Contemporary prevention strategies have achieved only modest long-term success despite substantial advances in nutritional science, suggesting that additional biological dimensions may require consideration [3,9]. Expanding the understanding of temporal nutritional physiology may therefore contribute to the development of more comprehensive preventive approaches capable of complementing traditional dietary recommendations [9,38]. Importantly, this perspective does not replace established principles of healthy nutrition but instead seeks to integrate emerging evidence regarding biological timing into existing metabolic frameworks [9,38].

Given the continuous expansion of chrononutrition research and the increasing recognition that metabolic responses are influenced by both dietary composition and temporal organization [9,10], a comprehensive synthesis of the available evidence has become timely. Integrating knowledge derived from circadian biology, pediatric endocrinology, nutritional physiology, and behavioral nutrition may provide a more complete understanding of how early-day eating patterns influence metabolic regulation during childhood [20,38]. Such integration may also facilitate the identification of conceptual gaps that can guide future experimental research and support the development and future validation of integrative conceptual models capable of advancing pediatric metabolic science [20,38].

Recent pediatric evidence has further strengthened the relevance of chrononutrition, with systematic reviews reporting significant associations between meal timing, breakfast patterns, and metabolic health in children and adolescents [44]. This manuscript was conducted as a narrative conceptual review rather than a systematic review. The objective was to synthesize current evidence from chronobiology, chrononutrition, pediatric endocrinology, and nutritional physiology into an integrative conceptual framework capable of generating biologically plausible hypotheses regarding pediatric metabolic regulation. Relevant literature was identified through searches of major biomedical databases (including PubMed, Scopus, and Google Scholar), with emphasis on recent systematic reviews, meta-analyses, clinical trials, observational studies, and landmark publications considered essential for providing physiological context. Accordingly, no formal study selection process, risk-of-bias assessment, or quantitative evidence synthesis was performed (Table 1).

**Table 1:** Scientific domains integrated into the Costa Reset Conceptual Model. EMW, Early-Day Metabolic Window.

Scientific domain	Principal evidence synthesized in this review	Contribution to the conceptual framework
Circadian biology	Endogenous circadian rhythms regulate glucose metabolism, endocrine signaling, energy homeostasis, and peripheral clock synchronization [7,16,20].	Establishes the biological timing that defines the physiological environment of the Early-Day Metabolic Window (EMW).
Chrononutrition	Meal timing influences postprandial metabolism, insulin sensitivity, substrate utilization, and metabolic flexibility independently of caloric intake alone [20,22,23].	Supports the concept that nutrient timing represents an independent determinant of metabolic regulation.
Breakfast research	Current evidence indicates that the metabolic effects of breakfast depend on meal composition, food quality, timing, and physiological context rather than breakfast consumption alone [33,40,44].	Supports the reinterpretation of breakfast as a physiological event instead of a culturally defined meal.
Endocrine physiology	Morning activation of cortisol secretion, gastrointestinal function, appetite regulation, and glucose homeostasis creates a coordinated endocrine environment after overnight fasting [16,22,31].	Provides the physiological basis for considering the EMW as a period of increased metabolic responsiveness.
Pediatric metabolism	Childhood is characterized by developmental plasticity, endocrine maturation, and unique metabolic regulation that differs from adults [3,20,29,44].	Justifies the pediatric focus of the proposed conceptual framework.
Behavioral physiology	Sleep regularity, chronotype, meal timing, fasting duration, and daily routines influence circadian alignment and metabolic regulation [20,29,38,45].	Highlights that behavioral factors interact with physiological mechanisms during the EMW.
Integrative conceptual framework	Evidence from the above domains supports a systems-based interpretation of pediatric metabolic regulation [20,38].	Integrates the above physiological domains into a unified conceptual framework represented by the Costa Reset Conceptual Model.

## Review

Integrating circadian biology into pediatric metabolic regulation

The evidence synthesized throughout the preceding sections suggests that pediatric metabolic regulation should no longer be interpreted through isolated physiological mechanisms but rather as the product of a highly integrated biological network in which circadian organization, endocrine signaling, dietary behavior, environmental exposures, and developmental physiology continuously interact [7,16]. Although each component has been extensively investigated, their collective influence on metabolic homeostasis has increasingly emerged as a major focus of contemporary chrononutrition research [20,38]. This systems-oriented perspective represents an important conceptual shift in nutritional science, moving beyond reductionist interpretations toward a more comprehensive understanding of metabolic regulation throughout childhood [16,20,38].

Traditional nutritional paradigms have primarily emphasized caloric balance and macronutrient composition as the principal determinants of metabolic health [9]. While these variables remain fundamental, accumulating evidence indicates that they do not fully explain the considerable interindividual variability consistently observed in metabolic responses to similar dietary interventions [7,20]. Experimental and clinical studies increasingly demonstrate that endocrine rhythms, sleep behavior, feeding schedules, physical activity, circadian alignment, and environmental timing collectively modulate nutrient utilization and energy metabolism [17,20,26], suggesting that metabolic regulation is influenced by both nutritional and temporal dimensions [7,9,20].

From a physiological perspective, circadian biology provides the temporal architecture through which metabolic processes are coordinated [7,12]. Rather than functioning as passive oscillators, circadian clocks actively anticipate predictable environmental and behavioral cues, synchronizing hormonal secretion, glucose homeostasis, lipid metabolism, mitochondrial activity, immune regulation, and energy expenditure according to the biological time of day [13,15,16]. This anticipatory organization allows metabolic pathways to optimize substrate utilization before environmental demands occur, thereby improving physiological efficiency and maintaining homeostasis under normal conditions [7,16].

Within this framework, food intake should be interpreted not only as a source of energy and nutrients, but also as a potent biological timing signal capable of entraining peripheral circadian oscillators [13,16,20]. Feeding behavior activates multiple endocrine, neural, gastrointestinal, and metabolic pathways simultaneously, triggering coordinated physiological responses that extend well beyond digestion [16,19]. These responses include pancreatic hormone secretion, hepatic glucose regulation, intestinal incretin release, autonomic nervous system activation, and tissue-specific metabolic adaptations, all of which operate under circadian modulation [19,20,22]. Consequently, identical meals may generate substantially different metabolic responses depending on the internal physiological environment in which they are consumed [20,22].

This concept becomes particularly relevant during childhood because pediatric metabolism is characterized by remarkable biological plasticity [3,28]. Ongoing maturation of endocrine systems, neural circuits, immune function, and metabolic pathways increases the responsiveness of children to environmental influences, including dietary habits and behavioral routines [3,28]. Repeated dietary exposures occurring during these developmental stages may therefore contribute to long-term metabolic programming through cumulative interactions between feeding behavior and endogenous biological rhythms [20,28,29]. Such observations reinforce the importance of considering temporal physiology when interpreting pediatric nutritional interventions [20,29].

Another important implication emerging from contemporary chronobiology is that metabolic regulation cannot be adequately understood by chronological time alone [18,20]. Clock time simply indicates when food is consumed, whereas biological time reflects the internal physiological state of the organism at the moment of nutrient exposure [20,22]. Factors such as sleep duration, circadian alignment, chronotype, previous fasting duration, hormonal oscillations, and habitual meal timing may substantially modify the biological context in which identical meals are metabolized [18,22,29]. This distinction provides a plausible explanation for the heterogeneous findings frequently reported in studies investigating breakfast consumption, meal timing, and obesity risk, where apparently contradictory results may instead reflect differences in biological timing rather than inconsistencies in nutritional physiology itself [29,40].

Endocrine regulation further illustrates the complexity of these interactions [7,16]. Hormones involved in energy homeostasis, including cortisol, insulin, glucagon, leptin, ghrelin, glucagon-like peptide-1 (GLP-1), glucose-dependent insulinotropic polypeptide (GIP), and melatonin, display pronounced circadian oscillations that continuously modify appetite regulation, insulin sensitivity, hepatic glucose production, gastric emptying, lipid utilization, and energy expenditure [16,20,31]. Consequently, nutrient metabolism occurs within a dynamic hormonal environment rather than under static physiological conditions, reinforcing the concept that feeding behavior should be interpreted within its broader endocrine and circadian context rather than as an isolated nutritional event [7,20,31].

Particular attention should be directed toward the physiological transition occurring immediately after the overnight fasting period [20,22]. This interval is characterized by coordinated activation of multiple metabolic systems as the body transitions from endogenous substrate production toward postprandial nutrient utilization [16,20]. Hepatic glucose output progressively declines, pancreatic endocrine activity increases, incretin secretion is stimulated, peripheral glucose uptake increases, and cellular energy metabolism adapts to the renewed availability of nutrients [22,30,31]. Rather than representing a simple digestive event, this transition constitutes a highly coordinated metabolic process involving simultaneous communication among the central nervous system, endocrine organs, gastrointestinal tract, liver, skeletal muscle, adipose tissue, and peripheral molecular clocks [16,20,22].

Collectively, these observations support the interpretation that pediatric metabolic health emerges from the continuous interaction among biological timing, endocrine physiology, dietary composition, behavioral routines, and developmental metabolism [7,16]. None of these variables appears to operate independently; instead, they form a dynamic physiological network whose efficiency depends on the degree of synchronization among its multiple regulatory systems [16,20]. Recognizing this biological integration provides the conceptual foundation for investigating whether specific periods of the daily metabolic cycle may represent phases of increased physiological sensitivity to nutritional exposures [20,38].

Rethinking the modern breakfast paradigm

For decades, breakfast has occupied a central position within nutritional science and public health recommendations [40]. Traditionally promoted as the "most important meal of the day," breakfast has been associated with improved dietary quality, enhanced cognitive performance, healthier body weight, and reduced cardiometabolic risk [33,41,42]. However, the growing body of evidence accumulated over the past decade suggests that this long-standing paradigm requires substantial reconsideration [33,40]. Rather than focusing exclusively on breakfast consumption per se, contemporary research increasingly indicates that its physiological effects depend on a complex interaction involving meal composition, degree of food processing, circadian alignment, behavioral context, and the internal metabolic state at the time of the first daily food intake [20,33,40]. Recent pediatric systematic evidence further supports this perspective by reporting consistent associations between breakfast patterns, meal timing, and metabolic health outcomes in children and adolescents [44].

This shift in scientific perspective reflects a broader evolution in nutritional physiology [20]. Early observational studies frequently categorized individuals simply as breakfast consumers or breakfast skippers, implicitly assuming that breakfast represented a homogeneous dietary behavior [33,40]. Such a classification is now recognized as an oversimplification [40]. Two individuals may both consume breakfast while being exposed to profoundly different metabolic conditions depending on meal timing, sleep duration, chronotype, dietary quality, glycemic characteristics, physical activity patterns, and habitual eating schedules [20,29]. Consequently, breakfast should no longer be interpreted as a discrete nutritional event but rather as an integrated physiological process whose metabolic consequences extend far beyond the first meal itself [20,29,33].

The composition of breakfast has also undergone substantial transformation during recent decades [32,33]. In many industrialized and developing countries, traditional breakfasts based on minimally processed foods have progressively been replaced by highly processed products characterized by refined carbohydrates, added sugars, industrial fats, low dietary fiber content, artificial additives, and high glycemic load [34,35,37]. This nutritional transition has occurred simultaneously with increasing consumption of sugar-sweetened beverages, ready-to-eat cereals, sweet bakery products, flavored dairy beverages, and convenience foods, particularly among children and adolescents [32,34]. Such changes suggest that the metabolic consequences attributed to breakfast cannot be adequately understood without considering food quality, degree of processing, and metabolic context [33-35,37].

Beyond nutritional composition, the physiological environment in which the first daily meal is consumed deserves equal consideration [20]. The period immediately following awakening represents a unique biological transition during which multiple endocrine and metabolic systems undergo coordinated activation [16,20]. Cortisol secretion reaches its morning peak, peripheral tissues progressively adjust insulin responsiveness, gastrointestinal motility resumes after prolonged fasting, hepatic glucose production begins to decline, and neural pathways involved in appetite regulation become increasingly active [22,31]. Rather than occurring in isolation, these processes collectively create a dynamic metabolic environment characterized by endocrine responsiveness and metabolic adaptability [16,20,31].

From this perspective, the first eating episode following the overnight fasting period may represent more than a simple interruption of fasting [20,22]. Nutrient exposure occurring during this interval interacts with endocrine oscillations, peripheral circadian clocks, gastrointestinal signaling, and substrate availability [22,30]. The resulting metabolic response therefore reflects not only the characteristics of the ingested meal but also the biological conditions under which nutrient processing is initiated [20,26,31]. This interpretation expands current chrononutrition paradigms by proposing that identical foods may elicit distinct physiological responses according to the biological and temporal context in which nutrient exposure occurs [20,22,26].

An additional consideration emerges from studies investigating postprandial metabolism and circadian metabolic physiology [20,22]. Glycemic excursions, insulin secretion, satiety signaling, substrate oxidation, and diet-induced thermogenesis all appear to exhibit circadian variation throughout the day [22,23,31]. Morning metabolic responses generally differ from those observed during evening hours, suggesting that nutrient metabolism is influenced by endogenous biological timing independently of caloric intake alone [20,22,23]. Although individual responses remain variable, these observations collectively support the concept that the physiological characteristics of the first daily eating episode may influence subsequent metabolic regulation through mechanisms that extend beyond the immediate postprandial period [20,22,31].

Importantly, these mechanisms should not be interpreted as evidence either supporting universal breakfast consumption or advocating the indiscriminate omission of the first daily meal [20,29,33]. Such binary interpretations oversimplify an inherently complex physiological phenomenon [40]. Instead, the available evidence suggests that the metabolic significance of the first daily meal depends on multiple interacting variables, including meal quality, meal timing, fasting duration, circadian alignment, behavioral regularity, sleep characteristics, and overall dietary pattern [20,29,33]. Consequently, discussions centered exclusively on whether breakfast should or should not be consumed fail to capture the biological complexity underlying early-day metabolic regulation [20,40].

These observations support the need for a broader conceptual framework capable of integrating temporal physiology with nutritional quality [20,38]. Rather than interpreting breakfast solely as a culturally defined meal, it may be more appropriate to consider the biological characteristics of the period during which the first nutritional exposure occurs [20,22]. This perspective shifts attention from the meal itself toward the physiological conditions present during the transition from overnight fasting to daytime metabolism [20,22,31]. This transition appears to represent a coordinated metabolic phase characterized by synchronized endocrine activation, circadian signaling, gastrointestinal adaptation, and progressive substrate utilization [16,20,22,31].

The Early-Day Metabolic Window (EMW)

Based on this interpretation, the present review proposes that this interval may be more appropriately conceptualized as the Early-Day Metabolic Window (EMW) [20,38]. We define the EMW as the physiological phase immediately following the overnight fasting state, in which circadian synchronization, endocrine responsiveness, metabolic flexibility, nutrient sensing, and peripheral clock coordination converge to shape metabolic regulation during the subsequent hours of the day [7,16,20,22]. Importantly, the EMW should not be understood as a fixed clock-time interval but rather as a biologically determined phase influenced by individual circadian organization, sleep-wake timing, fasting duration, and habitual behavioral patterns [18,22,29]. This distinction reinforces the concept that biological time may represent a more physiologically relevant determinant of metabolic regulation than chronological time alone [18,20,38]. Recent pediatric evidence also suggests that the temporal distribution and daily duration of eating may contribute to metabolic regulation during childhood, reinforcing the importance of considering biological timing together with eating patterns within the EMW [45].

The introduction of the EMW does not seek to replace existing chrononutrition principles but rather to provide an integrative physiological perspective capable of connecting evidence that has traditionally been examined in isolation [20,38]. Circadian biology explains temporal metabolic variation; endocrinology describes hormonal synchronization; chrononutrition investigates meal timing; nutritional science evaluates dietary quality; and pediatric metabolism highlights developmental plasticity [9,20,26]. However, relatively few conceptual frameworks have attempted to integrate these mechanisms into a unified model specifically addressing the metabolic significance of the first daily nutritional exposure during childhood [20,26,38].

Recognizing the EMW as a biologically distinct physiological phase serves as the conceptual bridge for integrating current evidence into a unified framework capable of explaining how the first daily nutritional exposure may influence pediatric metabolic regulation [20,38]. This integrative framework is presented in the following section as the Costa Reset Conceptual Model.

The Costa Reset Conceptual Model: An integrative framework for early-day metabolic regulation

Building on the evidence synthesized in this review, we propose the Costa Reset Conceptual Model, an integrative physiological framework that synthesizes current evidence from chronobiology, chrononutrition, pediatric endocrinology, nutritional physiology, and behavioral nutrition into a unified conceptual structure [20,38]. Rather than introducing a new biological mechanism, the model integrates established physiological principles to explain how temporal nutritional exposures interact with endogenous regulatory systems during a period of heightened metabolic responsiveness [16,20,38]. In doing so, it provides a systems-based interpretation of pediatric metabolic regulation in which biological timing, endocrine signaling, nutrient sensing, dietary quality, and developmental physiology function as interconnected components of a dynamic physiological network [7,16,38].

The Costa Reset Conceptual Model is based on the premise that the EMW represents a biologically distinct phase during which multiple regulatory systems simultaneously transition from the overnight fasting state toward active nutrient metabolism [20,38]. During this interval, circadian synchronization, hormonal oscillations, gastrointestinal activation, peripheral clock entrainment, glucose regulation, and nutrient sensing occur in close physiological coordination [16,20,22,31]. Because these physiological systems interact simultaneously and continuously rather than functioning as isolated mechanisms, nutritional exposures occurring during the EMW may generate coordinated physiological responses capable of influencing metabolic regulation beyond the immediate postprandial period [16,20,22,26].

Within the Costa Reset Conceptual Model, the first daily nutritional exposure is interpreted as a physiological signal capable of interacting with ongoing endocrine and circadian processes [20,22]. Nutrient quality, degree of food processing, glycemic characteristics, meal timing, and behavioral regularity collectively determine the biological information delivered to metabolically active tissues during this transitional phase [30,34]. Consequently, metabolic responses arising during the EMW are proposed to reflect not only the nutritional characteristics of the ingested meal but also the degree of physiological synchronization between feeding behavior and endogenous biological rhythms [20,22,26].

A defining characteristic of the Costa Reset Conceptual Model is its emphasis on physiological integration rather than reductionist nutritional interpretation [20,38]. Traditional nutritional models often emphasize isolated variables, including total caloric intake, macronutrient distribution, glycemic index, or meal frequency [7]. Although these variables remain clinically relevant, they may inadequately represent the complexity of metabolic regulation when interpreted independently [7,16]. The Costa Reset Conceptual Model proposes that metabolic regulation emerges from the continuous interaction among biological timing, endocrine responsiveness, dietary quality, environmental exposures, behavioral consistency, and developmental physiology, thereby providing a multidimensional framework for interpreting pediatric metabolic regulation [16,20,38].

This systems-based approach is consistent with the growing recognition that metabolic regulation emerges from the dynamic interaction among multiple regulatory systems rather than from the isolated behavior of individual mechanisms [7,16]. Circadian rhythms coordinate hormonal secretion; endocrine responses regulate substrate utilization; nutrient availability influences peripheral molecular clocks; behavioral routines modify circadian alignment; and repeated nutritional exposures contribute to long-term metabolic adaptation [16,20]. Collectively, these interconnected mechanisms suggest that early-day nutritional behavior may represent a critical point of physiological convergence within pediatric metabolic regulation rather than merely another daily eating occasion [20,38].

Importantly, the Costa Reset Conceptual Model should not be interpreted as advocating universal breakfast consumption, prolonged fasting, or any rigid dietary protocol [20,33,40]. Instead, it emphasizes the physiological characteristics of the metabolic environment during the EMW and proposes that optimizing the physiological conditions present during this period may represent an additional dimension of personalized nutritional care [20,26]. Accordingly, the model shifts the scientific discussion away from simplistic dichotomies such as "breakfast versus breakfast skipping" toward a more comprehensive understanding of how biological timing, dietary quality, and physiological context interact to influence pediatric metabolic regulation [20,33,40].

Another important characteristic of the Costa Reset Conceptual Model is its compatibility with current chrononutrition research [9,20]. Rather than replacing or contradicting established concepts such as time-restricted eating, early time-restricted feeding, circadian alignment, or behavioral nutrition, the model integrates these principles into a broader physiological framework [20,23,26]. Accordingly, it should be viewed as complementary rather than competitive, providing an integrative conceptual framework through which previously independent observations can be interpreted within a unified physiological context. This integrative perspective broadens the model's potential applicability across clinical, behavioral, and research settings while remaining consistent with the current body of scientific evidence [9,20,38].

The Costa Reset Conceptual Model also provides a biologically plausible framework for understanding the heterogeneity frequently observed in nutritional intervention studies [17,20]. Individuals receiving apparently similar dietary recommendations often exhibit markedly different metabolic responses despite comparable caloric intake [17,23]. Variability in circadian alignment, sleep quality, chronotype, endocrine oscillations, habitual meal timing, and the physiological characteristics of the EMW may partially explain these differences [20,23,29]. Although this interpretation remains hypothetical and requires prospective experimental validation, it provides a biologically plausible basis for generating testable hypotheses and guiding future pediatric chrononutrition research [20,38].

From a translational perspective, the Costa Reset Conceptual Model encourages a gradual shift from static nutritional prescriptions toward chronobiologically informed nutritional strategies [9,20]. Such an approach does not replace established dietary recommendations but rather expands their physiological rationale by incorporating biological timing as an additional dimension of metabolic regulation [20,26]. Consequently, nutritional counseling may eventually evolve from focusing exclusively on what children eat to also considering when nutrients are consumed, the physiological conditions under which they are consumed, and the biological context in which nutrient exposure occurs [20,26,38].

The principal contribution of the Costa Reset Conceptual Model lies not in proposing a new dietary intervention but in providing an integrative physiological framework that connects chronobiology, chrononutrition, endocrine physiology, developmental metabolism, and dietary quality within a unified conceptual structure [9,20]. By integrating these previously fragmented areas of knowledge, the model offers a coherent physiological rationale for investigating how nutritional exposures occurring during the EMW may influence pediatric metabolic health [20,38]. More importantly, it establishes a theoretical foundation for future experimental studies designed to evaluate the clinical relevance of this integrative physiological perspective [20,38].

Clinical implications, limitations, and future perspectives

The growing global burden of childhood obesity reinforces the need for innovative approaches capable of complementing current evidence-based nutritional strategies [3]. Despite substantial advances in nutritional science, achieving sustainable long-term prevention and treatment remains challenging, suggesting that additional physiological dimensions should be incorporated into pediatric nutritional care [3,9]. Within this context, the Costa Reset Conceptual Model offers an integrative physiological framework by emphasizing that dietary interventions may benefit not only from improvements in food quality but also from greater consideration of biological timing and circadian physiology [20,38]. Rather than replacing current evidence-based nutritional recommendations, the model seeks to expand their physiological rationale by integrating biological timing and temporal organization into the assessment of pediatric metabolic health [9,20,38].

From a clinical perspective, the Costa Reset Conceptual Model encourages healthcare professionals to evaluate pediatric eating behaviors beyond conventional nutritional parameters [20,38]. The assessment of pediatric patients could increasingly incorporate chronobiological and behavioral variables, including meal regularity, sleep consistency, circadian alignment, fasting duration, and the nutritional characteristics of the first daily eating episode [20,29]. Although these variables are frequently evaluated independently in clinical practice, their combined physiological interaction may provide a more comprehensive understanding of pediatric metabolic regulation [3,20,29]. Recent pediatric evidence further indicates that later meal timing may be associated with less favorable metabolic profiles, including greater insulin resistance among children and adolescents, supporting the clinical relevance of evaluating meal timing alongside dietary quality [46]. Such an integrative approach may facilitate more individualized nutritional counseling while remaining fully compatible with established evidence-based pediatric dietary guidelines [3,38].

The Costa Reset Conceptual Model may also provide a conceptual framework for public health initiatives aimed at childhood obesity prevention [3,38]. Contemporary nutritional education programs traditionally emphasize healthy food selection, portion control, and the reduction of ultra-processed food consumption [3,34]. Although these recommendations remain fundamental, integrating chronobiological principles into nutritional education may further strengthen preventive strategies by encouraging greater synchronization between daily behavioral routines and endogenous physiological rhythms [9,38]. Educational interventions promoting regular sleep schedules, consistent meal timing, and improved quality of the first daily nutritional exposure may represent valuable complementary components of future pediatric health promotion strategies [3,9,38].

Another important contribution of the Costa Reset Conceptual Model lies in its potential to support translational research [20,38]. By integrating circadian biology, endocrinology, chrononutrition, and pediatric metabolism into a unified physiological framework, the model generates multiple biologically plausible and experimentally testable hypotheses [20,26,38]. Rather than representing a definitive explanation of pediatric metabolic regulation, it should be viewed as a conceptual framework capable of guiding future investigations into the interaction between biological timing and nutritional behavior [20,38]. This characteristic is particularly important because comprehensive reviews are expected not only to synthesize existing evidence but also to identify conceptual gaps that stimulate future scientific investigation [20,38].

Nevertheless, several limitations must be acknowledged. First, the Costa Reset Conceptual Model is inherently conceptual and should not be interpreted as a clinically validated therapeutic intervention or evidence-based clinical strategy [20,38]. Although each physiological component incorporated into the model is individually supported by previously published evidence, the proposed integrative framework has not yet been prospectively evaluated [20,38]. Consequently, any causal relationship between optimization of the EMW and improvements in pediatric metabolic health remains hypothetical and requires rigorous prospective experimental validation.

A second important limitation concerns the current evidence base supporting chrononutrition research [20,38]. Most available evidence has been generated in adult populations, whereas pediatric investigations remain comparatively scarce [20,33]. Children exhibit unique developmental characteristics, endocrine profiles, growth trajectories, and behavioral patterns that may influence circadian physiology differently from those observed in adults [3,33]. Accordingly, direct extrapolation of findings from adult populations to pediatric clinical practice should be approached with caution until age-specific evidence becomes available [3,20,38].

Methodological heterogeneity represents another major limitation of the current evidence base [20,38]. Studies investigating breakfast consumption, meal timing, chronotype, chrononutrition interventions, and circadian alignment frequently employ different definitions, outcome measures, and methodological approaches, making direct comparison across studies inherently challenging [20,29,33]. Such heterogeneity complicates the interpretation of available evidence [20,29]. This limitation reinforces the need for standardized definitions, harmonized outcome measures, and prospective study designs in future pediatric chrononutrition research [20,29,38].

Future research should prioritize well-designed prospective pediatric cohort studies investigating temporal dietary patterns across different stages of childhood [20,38]. Randomized controlled trials examining interventions targeting early-day eating behavior while simultaneously monitoring glycemic regulation, insulin sensitivity, inflammatory biomarkers, appetite-regulating hormones, body composition, and circadian biomarkers would provide important evidence regarding the physiological relevance of the EMW [20,23,26]. In addition, continuous glucose monitoring, metabolomic profiling, circadian hormone assessment, wearable-based sleep monitoring, and other digital health technologies may further elucidate the biological mechanisms proposed within the Costa Reset Conceptual Model [20,26,38].

Another important research priority is to investigate whether individual biological and behavioral characteristics modify responses to early-day nutritional interventions [20,38]. Chronotype, sleep duration, pubertal maturation, genetic variability in circadian clock genes, gut microbiota composition, habitual physical activity, and family behavioral routines may all influence the physiological characteristics of the EMW [20,29]. Identifying these potential modifiers may support the development of personalized chrononutrition strategies while enhancing our understanding of the interindividual variability frequently observed in metabolic intervention studies [20,29,38].

Finally, the Costa Reset Conceptual Model should be regarded as a dynamic conceptual framework rather than a static theoretical construct [20,38]. As new evidence emerges, the model's physiological components may be refined, expanded, or modified [20,38]. This adaptability represents one of the model's principal strengths, allowing it to evolve alongside advances in chronobiology, nutritional physiology, pediatric endocrinology, and related biomedical sciences [20,38]. Ultimately, the scientific value of the Costa Reset Conceptual Model will depend not on the originality of any individual concept, but on its capacity to integrate existing evidence, generate biologically plausible and clinically relevant hypotheses, and stimulate high-quality experimental research capable of advancing our understanding of pediatric metabolic regulation [20,38].

## Conclusions

Childhood obesity remains one of the most important public health challenges worldwide, and growing evidence indicates that pediatric metabolic regulation is influenced not only by dietary composition but also by biological timing. The evidence synthesized in this review supports the interpretation that pediatric metabolic regulation emerges from the continuous interaction among circadian organization, endocrine signaling, dietary quality, feeding behavior, and developmental physiology. Within this context, the EMW is proposed as a physiologically relevant framework for understanding how the transition between overnight fasting and the first daily nutritional exposure may influence metabolic regulation during childhood.

Building upon this perspective, the Costa Reset Conceptual Model is presented as an integrative physiological framework that synthesizes current evidence from chronobiology, chrononutrition, pediatric endocrinology, and nutritional physiology. Rather than proposing a new biological mechanism or dietary intervention, the model provides a conceptual structure for interpreting how biological timing and nutritional exposures may interact during the early hours of the day. Although the model remains conceptual and requires prospective validation, it offers biologically plausible and testable hypotheses that may guide future pediatric research. By integrating previously fragmented areas of knowledge, the Costa Reset Conceptual Model contributes to the advancement of pediatric chrononutrition and provides a theoretical foundation for future investigations into temporal nutritional physiology and childhood metabolic health.
